# Development a m^6^A regulators characterized by the immune cell infiltration in stomach adenocarcinoma for predicting the prognosis and immunotherapy response

**DOI:** 10.18632/aging.204574

**Published:** 2023-03-17

**Authors:** Guodong Shi, Yang Li, Huijiang Gao, Yucheng Wei, Yuanyong Wang

**Affiliations:** 1 Department of Thoracic Surgery, Affiliated Hospital of Qingdao University, Qingdao, China; 2 Department of Obstetrics, Affiliated Hospital of Qingdao University, Qingdao, China; 3 Department of Thoracic Surgery, Tangdu Hospital of Air Force Military Medical University, Xi’an, China

**Keywords:** STAD, m^6^A regulator, TIME, m^6^A score, therapy

## Abstract

N6-Methyladenosine (m^6^A) has attracted growing interest among scholars as an important regulator of mRNA expression. Although the significant role of m^6^A in multiple biological processes (like proliferation and growth of cancers) has been comprehensively described, an analysis of its possible role in stomach adenocarcinoma (STAD) of tumor immune microenvironment (TIME) remains lacking. The data for RNA expression, single nucleotide polymorphism (SNP), and copy number variation (CNV) were downloaded from The Cancer Genome Atlas (TCGA). Subsequently, 23 m^6^A regulators were curated, with patients being clustered into three m^6^A subtypes and m^6^A-related gene subtypes. Furthermore, they were compared based on overall survival (OS). This study also evaluates the association between m^6^A regulators and immune as well as response to the treatment. According to the TCGA-STAD cohort, three m^6^A clusters conformed to three phenotypes, immune-inflamed, immune-dessert, and immune-excluded, respectively. Patients who displayed lower m^6^A scores presented better overall survival outcomes. The GEO cohort demonstrated that those with a low m^6^A score had obvious general survival benefits and clinical advantages. Low m^6^A scores can carry the enhanced neoantigen loads, triggering an immune response. Meanwhile, three anti-PD-1 cohorts have confirmed the value of predicting survival outcomes. The results of this study indicate that m^6^A regulators are associated with TIME, and the m^6^A score is an efficient prognostic biomarker and predictive indicator for immunotherapy and chemotherapeutics. Moreover, comprehensive evaluations of m^6^A regulators in tumors will broaden our comprehension of TIME, efficiently guiding enhancing explorations on immunotherapy and chemotherapy strategies for STAD.

## INTRODUCTION

Stomach adenocarcinoma (STAD) is one of the most common pathological types of gastric cancer, the sixth most normal malignancy worldwide, and the fourth major reason for tumor-related deaths [1]. Despite obvious development in the diagnosis and treatment for STAD, the prognosis for patients with STAD remains poor due to postoperative recurrences and advanced stages [2, 3]. Thus, identifying novel effective biomarkers for early detection and therapeutic targets for patients with STAD is crucial.

Several studies indicate that N6-methyladenosine (m^6^A) plays an essential role in tumorigenesis and the development of various cancers, including STAD [4, 5]. As a dynamic process, m^6^A RNA modification is primarily regulated via adenosine methyltransferases (“writers”) and demethylases (“erasers”) and performs specific functions through interacting with m^6^A binding proteins (“readers”). m^6^A is widely distributed in various RNAs, such as messenger RNA (mRNA), pre-microRNA (pri-miRNA), circular RNA (circRNA), and lncRNA. Moreover, m^6^A is associated with tumorigenesis and development, including STAD [6–8]. For example, the m^6^A writer METTL3-catalyzed m^6^A modification was found to stimulate the expression of NOTCH1 and activate the Notch signaling pathway in esophageal squamous cell carcinoma [9]. METTL3 might stimulate m^6^A modification of HDGF expression, and the m^6^A reader IGF2BP3 could increase its stability, which recognizes and binds to the m^6^A site to promote tumor angiogenesis and glycolysis in STAD [10]. Furthermore, previous literature proved that m^6^A plays a critical role in the tumor microenvironment (TME), immune recognition, and immune response [11–13].

Recently, although increasing evidence indicates a correlation between m^6^A modification and immune cell infiltration, the cancer-related pathways of m^6^A methylation in tumor immune microenvironment (TIME) are still little understood. According to Han et al., lysosomal proteases labeled and identified by YTHDF1 can trigger the degradation of tumor neoantigens [14]. Moreover, they found higher expression levels of NK cells and CD8+ cytotoxic T cells in the tumors of YTHDF1 knockout mice than WT mice, suggesting a stronger anti-tumor response when YTHDF1 is present. Chong et al. pointed out that interferon-gamma (IFN-γ)-induced cytotoxicity in melanoma cells was capable of degrading through FTO *in vitro* by lowering the levels of cell-intrinsic genes PD-1, SOX10, and CXCR4 expression which through YTHDF2-mediated decay [15]. However, due to technical limitations, almost all research has focused on one or two m^6^A regulators. Therefore, a comprehensive investigation of multiple m^6^A regulators in STAD, including the associations between m^6^A regulators and CNVs and TMBs, as well as the prognostic value and the risk score in immunotherapy and chemotherapy, will provide a more comprehensive understanding of the TIME.

In this study, we screened the Cancer Genome Atlas (TCGA) database for m^6^A-related genes associated with STAD, thereby assessing the correlation between m^6^A methylation and prognosis, CNVs, TMB, and TIME of STAD. Subsequently, we identified three clustering subtypes through the “Consensus Cluster Plus” method, and the above three subtypes were closely related to the following three phenotypes, respectively, immune-inflamed, immune excluded, and immune-desert [16]. Afterward, a scoring model, m^6^A score, was constructed to quantify STAD of individual samples. This study explored the correlations between carcinogenic pathways and ICI treatment, scoring model, and TIME to discover the influence of m^6^A regulators in STAD. Herein, it is demonstrated that m^6^A regulators play an indispensable role in TIME and contribute to making therapeutic strategies on STAD.

## MATERIALS AND METHODS

### The collection and pretreatment of datasets and samples

In this study, the genomics data and clinical information of STAD patients were obtained from the public TCGA database (https://cancergenome.nih.gov/), containing 350 tumor samples and 32 normal samples. The selection criteria were applied as 1) Complete clinical and OS information, including gender, age, stage, and radiation therapy, were collected for the investigation. 2) Histologically confirmed STAD. The UCSC Xena (https://gdc.xenahubs.net/) obtains mutation data. Twenty-three m^6^A regulators were gained as per previous research (Supplementary Table 1). The number of nonsynonymous and synonymous mutations was stated as the tumor mutation burden. The GSE84437 (N = 433) from Gene Expression Omnibus (GEO https://www.ncbi.nlm.nih.gov/geo/) datasets were used as the validation cohort (Table 1).

**Table 1:** Comparison of clinical-pathological characteristics between TCGA-STAD cohort and GSE84437 cohort.

	**TCGA-STAD cohort n=350**	**GSE84437 cohort n=433**	**P**
Age, median [min, max]	67.0 [35.0, 90.0]	62.0 [27.0, 86.0]	0.002
≤60	117(33.4%)	194(44.8%)	
>60	230(65.7%)	239(55.2%)	
Unknown	3(0.8%)	0(0)	
Gender (n, %)			0.263
Male	226(64.6%)	296(68.4%)	
Female	124(35.4%)	137(31.6%)	
OS Status (n, %)			0.061
Alive	204(58.3%)	224(51.7%)	
Dead	145(41.4%)	209(48.2%)	
Unknown	1(0.3%)	0(0)	
OS time (months), median [min, max]	15.8 [0.03, 124]	70.0 [1.00, 161]	<0.001
T (n, %)			
T1	16(4.6%)	11(2.5%)	
T2	74(21.1%)	38(8.8%)	
T3	161(46.0%)	92(21.2%)	
T4	95(27.1%)	292(67.4%)	
Unknown	4(1.1%)	0(0)	
N (n, %)			<0.001
N0	103(29.4%)	80(18.5%)	
N1	93(26.6%)	188(43.4%)	
N2	72(20.6%)	132(30.5%)	
N3	71(20.3%)	33(7.6%)	
Unknown	11(3.1%)	0(0)	
M (n, %)			-
M0	312(89.1%)	NA	
M1	23(6.6%)	NA	
Unknown	15(4.3%)	NA	
TNM stage (n, %)			-
I	46(13.1%)	NA	
II	110(31.4%)	NA	
III	145(41.4%)	NA	
IV	35(10.0%)	NA	
Unknown	14(4.0%)	NA	
Tumor Grade (n, %)			-
G1	9(2.6%)	NA	
G2	125(35.7%)	NA	
G3	207(59.1%)	NA	
Unknown	9(2.6%)	NA	

### The consensus clustering of 23 m^6^A regulators by consensus cluster plus

We employed the Consensus Cluster Plus R package to elucidate the biological role of m^6^A regulators in STAD categorize patients into different m^6^A isoforms [17]. “PCA” package was used to analyze gene expression among distinct m^6^A subtypes.

### Gene set variation analysis (GSVA)

The biological processes among disparate m^6^A subtypes were studied using the GSVA package [18]. We used the MSigDB and IMvigor210 CoreBiologies packages to enrich well-defined biological pathways and roles [19, 20]. The Gene Ontology (GO) annotation of m^6^A-related genes was carried out using the “ClusterProfiler” package [21].

### Immune-related function and immune cell infiltration estimation via ssGSEA

We estimated the proportions of the 23 tumor-infiltrating immune cell types from each sample using the “CIBERSORT” package based on the STAD gene expression matrix. Furthermore, the association between immune-related pathways and different m^6^A subtypes was discovered by ssGSEA in TCGA-STAD profiles.

### Immune response analysis

Based on the immunophenoscore (IPS) of 415 STAD patients, we created the Cancer Immunome Database (TCIA) (https://www.tcia.at/home), which could provide a comprehensive analysis of immunogenomic data. Furthermore, this study employed the Tumor Immune Dysfunction and Exclusion (TIDE) method to predict immune checkpoint blockade (ICB) response and tumor immune evasion mechanisms. The ESTIMATE package was used to assess tumor purity and tumor cellularity based on expression matrixes composed of the TIME. In addition, the summation of the immune and stromal scores from each sample was defined as the tumor purity. According to the tumor sample, which contains lower tumor purity and higher immune scores, an abundance of immune cells may have infiltrated the tumor.

### DEGs associated among the m^6^A phenotypes

According to the consensus clustering algorithm, patients were divided into three m^6^A clusters and identified as differentially expressed genes (DEGs). The “limma” package was used to verify DEGs among three m^6^A clusters [22]. The screening criteria were well-defined as *p*-value <0.01.

### Evaluation of the m^6^A gene signature

The intersected DEGs were derived from the univariate Cox regression. The number of gene clusters was then determined by adopting the consensus clustering algorithm. The prognosis-related genes were discovered to construct Principal Component Analysis (PCA) and extracted the m^6^A score [23, 24]. The advantage of this algorithm is that it focuses mainly on negatively (or positively) correlated genes. The m^6^A score formula is as follows:


$${\text{m}}^{6}\text{A}\;\text{score}=\Sigma (\text{PC}1_{\text{i}})+\Sigma (\text{PC}2_{\text{i}})$$

Lastly, the correlation between m^6^A score and TMB was performed via Spearman’s approach according to survival curve and synonymous and nonsynonymous mutation counts.

### Biological pathways with m^6^A score

There is evidence that a panel of signatures associated with distinct biological pathways, including: 1) epithelial mesenchymal transition (EMT); 2) immune-checkpoint; 3) pan-fibroblast TGFb response signature; 4) CD8 T-effector signature; 5) homologous recombination; 6) Fanconi anemia pathway; 7) WNT target; 8) base excision repair; 9) mismatch repair; 10) DNA damage repair; 11) DNA replication; 12) nucleotide excision repair; 13) cell cycle regulation; 14) antigen processing; 15) cell cycle and 16) FGFR3-related genes [20, 25].

### The immune-checkpoint cohorts analysis

Three independent anti-PD-L1 cohorts that could apply to genomic and clinical information were acquired from the IMvigor210 cohort [20] and GSE78220 cohort [26] to calculate the predictive value of the m^6^A score for immunotherapy.

### Evaluation of the sensitivity of chemotherapeutic drugs

Genomics of Drug Sensitivity in Cancer (GDSC) is the largest public pharmacogenomics database that could predict sensitivity between high and low m^6^A score subtypes [27]. The pRRophetic package could process the half-maximal inhibitory concentration (IC_50_) via the ridge regression model [28].

### Immunohistochemistry (IHC)

IHC experiment detected IRGs protein levels in STAD and corresponding normal tissues. First, each sample was subjected to 10% formalin fixation, paraffin embedding, and processing up to 4-μm consecutive sections. After that, the sections were treated with methanol, followed by BSA incubation and primary antibody staining. Then, we stained the samples with a secondary antibody after washing with PBS. Finally, each section was observed and photographed using a microscope. The primary antibodies were obtained from Abcam.

### Statistical analyses

R software (version 3.6.3) was applied for this study. Wilcoxon test was computed using the difference comparison of the DEGs between high and low m^6^A score groups. Association between tumor-infiltrating immune cells and m^6^A score was applied for Spearman’s correlation. Kaplan-Meier methods were visualized for the difference in overall survival (OS) between the high-risk and low-groups. In addition, the screening criterion was *p*-value <0.01.

### Data availability statement

Publicly available datasets were analyzed in this study. The datasets generated during the current study are available in the Cancer Genome Atlas (TCGA) public dataset (https://portal.gdc.cancer.gov/) and the Gene-Expression Omnibus (GEO) public dataset (https://www.ncbi.nlm.nih.gov/geo/).

## RESULTS

### Genetic expression of 23 m^6^A regulators in STAD

**Figure 1:**
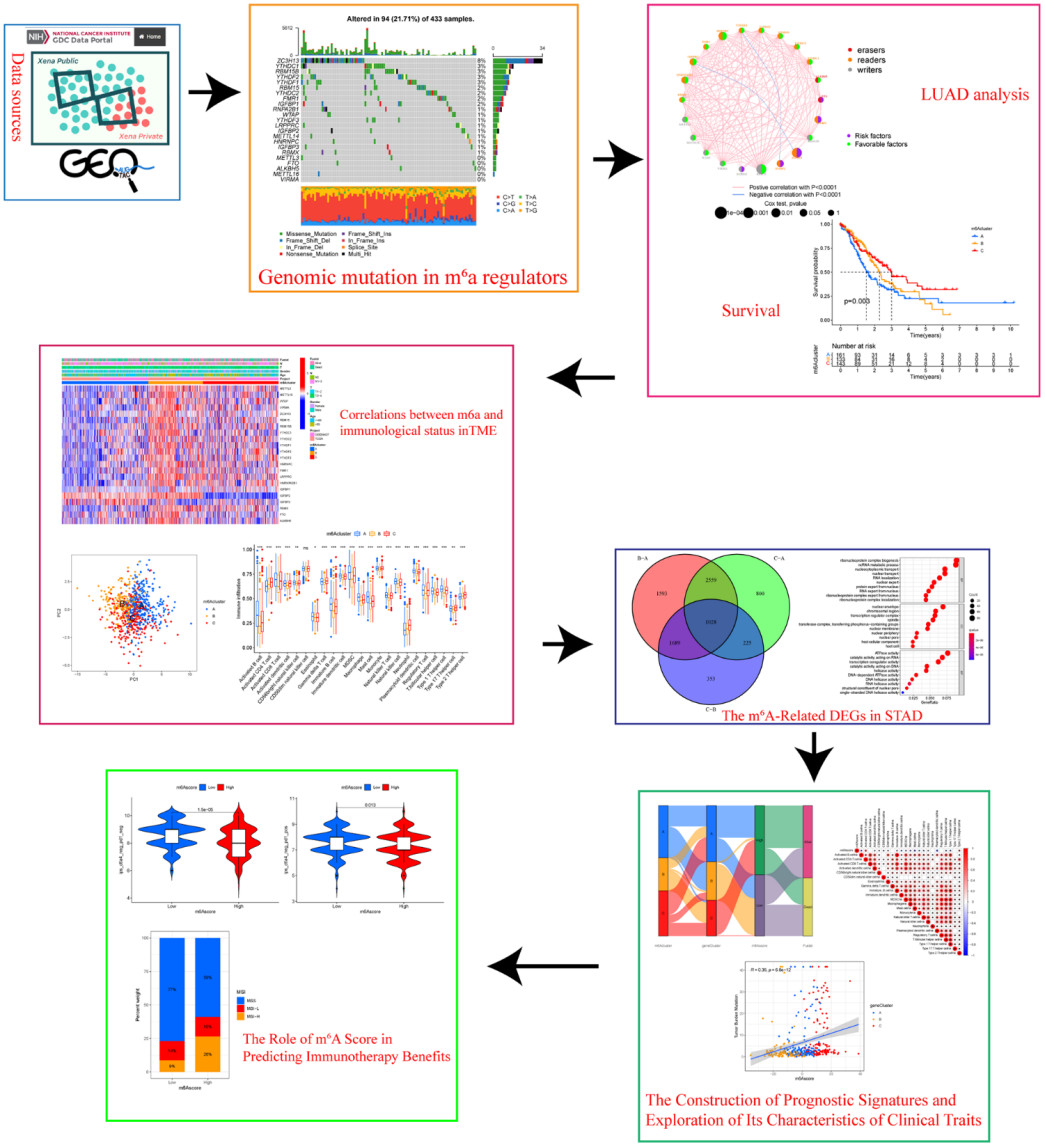
Research flow chart.

**Figure 2:**
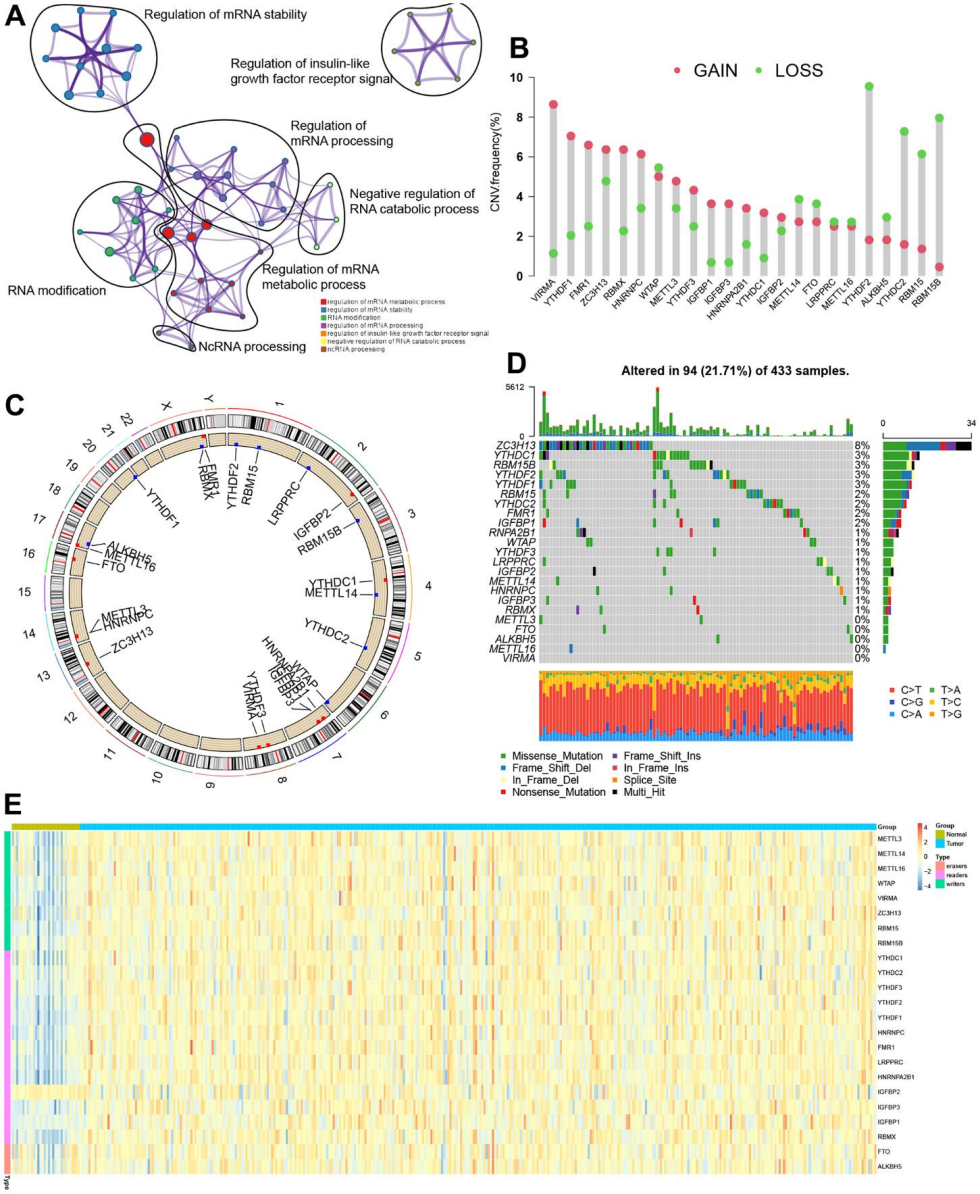
**Genetic topography of the 23 m^6^A regulators in STAD.** (**A**) Metascape network of 23 functionally enriched m^6^A regulators. Annotations denoted by different circles varied. (**B**) CNV map for 23 m^6^A regulators, where the column stood for the alteration frequency, and the green and pink dots, respectively, indicated the deletion and amplification of CNV. (**C**) CNV alteration sites for the cellular m^6^A regulators. (**D**) Among 433 patients, varying genetic alterations were noted in 94 patients, such as missense, nonsense, and splice-site mutations. (**E**) The different expression levels of 23 m^6^A regulators between normal and STAD.

Figure 1 shows the flow chart of this study. The TCGA dataset identified 23 m^6^A regulators (eleven “readers,” eight “writers,” and two “erasers”), which were summarized the biological functions and processes via the Metascape database (Figure 2A). Figure 2B shows the prevalent deletions of m^6^A regulators in copy number, while VIRMA, YTHDF1, and FMR1 featured an overall frequency of CNV amplification. Subsequently, as per Figure 2C, the location of CNV of all m^6^A regulators was listed in the chromosomes. Figure 2D shows that ZC3H13 showed the highest mutation frequency (8%), followed by RBM15B and YTHDC1, whereas VIRMA, METTL16, ALKBH5, FTO, and METTL3 did not display any mutations. Based on our analysis of the most mutated ZC3H13, nine of the other 22 m^6^a regulators are of interest (Supplementary Figure 1). Further investigation demonstrated that the expression of most m^6^a genes was significantly up-regulated in tumor samples except IGFBP2 (Figure 2E). m^6^A regulators (like VIRMA, YTHDF1, FMR1, and ZC3H13) with amplificated CNV showed higher expression than normal tissues. (Figure 2B, 2E). Following the obtained findings, we could demonstrate that m^6^A regulators exhibited significant transcriptome-altering landscapes and heterogeneity in genomics between STAD and normal samples.

### Identification of m^6^A subgroups mediated via 23 m^6^A regulators

**Figure 3:**
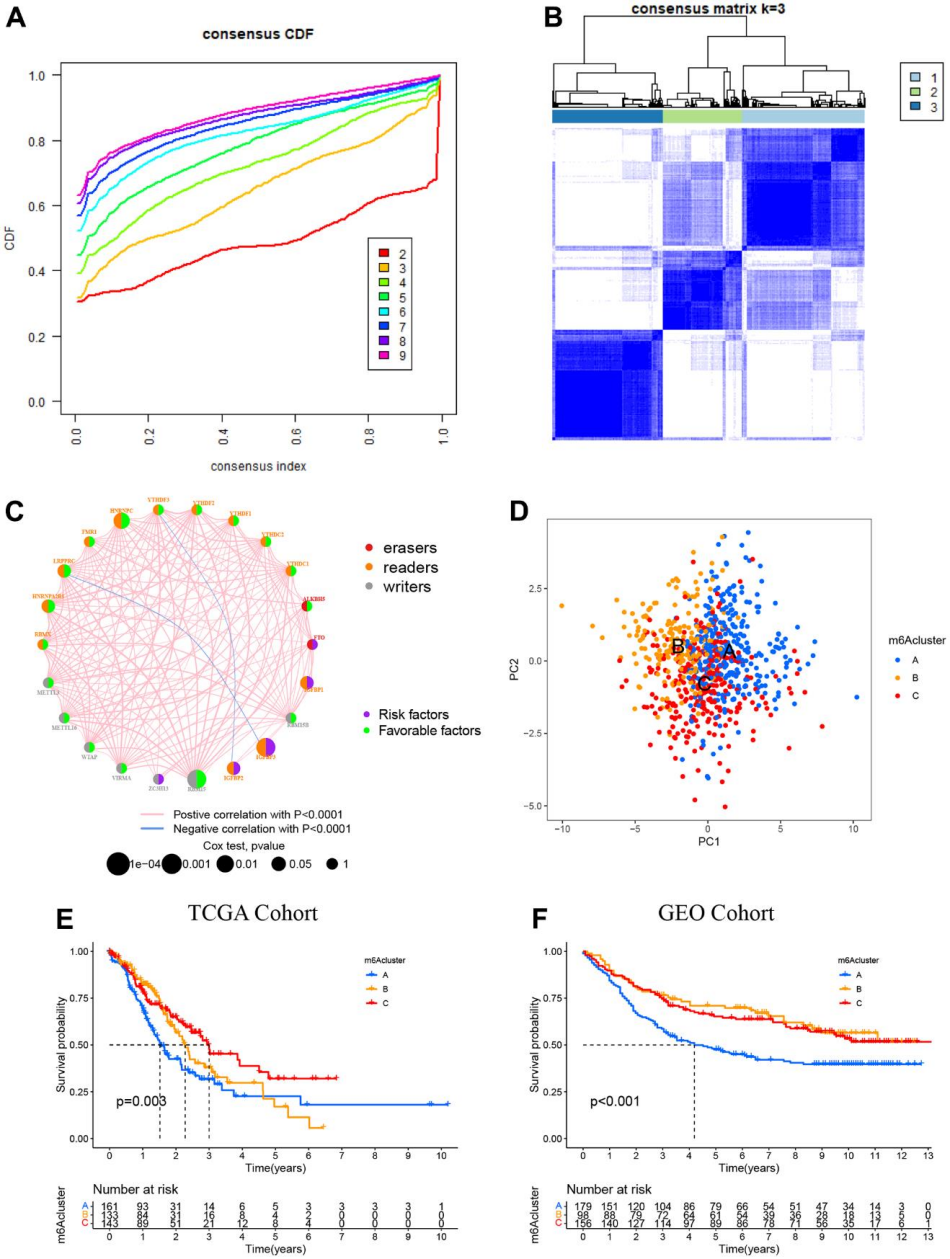
**Patterns of m^6^A methylation modification.** (**A**) CDF (cumulative distribution function; k = 2–9) in the right panel. (**B**) Depending on the consensus clustering matrix (k = 3), the patients with STAD were classified into three clusters. (**C**) Interactions among 22 m^6^A regulators in STAD. Circles in varying colors were used to represent differing RNA modifications, where red indicated Erasers, orange indicated readers, and gray indicated writers. Besides, green and purple circles, respectively, referred to the favorable and risky factors. (**D**) PCA (principal component analysis)-based map depicting prominent differences among the three m^6^A clusters. (**E**) Kaplan–Meier OS (overall survival) plots of 3 m^6^A clusters for the TCGA cohort (p = 0.003). (**F**) Kaplan–Meier OS plots of three m^6^A clusters for the GEO cohort (p < 0.001). OS was worse among those in the m^6^A cluster C as compared to the other 2 clusters.

The TCGA-STAD dataset with available clinical and survival information was enrolled into the training cohort. By applying the R package “Consensus ClusterPlus”, 350 STAD patients were stratified into three distinct following the expression of 23 m^6^A genes (Figure 3A, 3B, and Supplementary Figure 2). Variable selection was performed by LASSO Cox regression and the parameter λ indicated that the most suitable model to predict survival included RBM15, IGFBP1, RBMX, FTO, and ALKBH5 with coefficients of -0.306, 0.125, 0.042, 0.543, and -0.293, respectively (Supplementary Figure 3). The regulator network comprehensively represented the prognostic significance of 23 m^6^A regulators and their whole interactions (Figure 3C, and Supplementary Figure 4). FTO was found to be a risk factor and a favorable factor for ALKBHS in the eraser gene, and all other writers, except for IGFBP2 and IGFBP3 genes, were also favorable. In addition, we found that the 23 m^6^A regulators are positively correlated except for IGFBP2 and IGFBP3. Based on these results, the crosstalk among the 23 m^6^A regulators might play a key role in the pathogenesis and progression of individual tumors and in the formation of distinct m^6^A modifications. Thus, unsupervised clustering was adopted to classify samples into distinct m^6^A clusters. Furthermore, we could distinguish one m^6^A cluster from others completely in line with PCA (Figure 3D). Accordingly, three distinct m^6^A clusters were ultimately detected in the TCGA dataset, containing 161 in m^6^A cluster A, 133 in m^6^A cluster B, and 143 in m^6^A cluster C (Figure 3E). Among the above clusters, m^6^A cluster A, m^6^A cluster B, and m^6^A cluster C, m^6^A cluster A showed poor prognosis in the TCGA cohort, whereas patients in the m^6^A cluster C had an advantage in overall survival (p = 0.003). Similar results could also be found in the validation cohort (GEO cohort (Figure 3F).

### The distinct immune landscapes of TIME in m^6^A clusters

**Figure 4:**
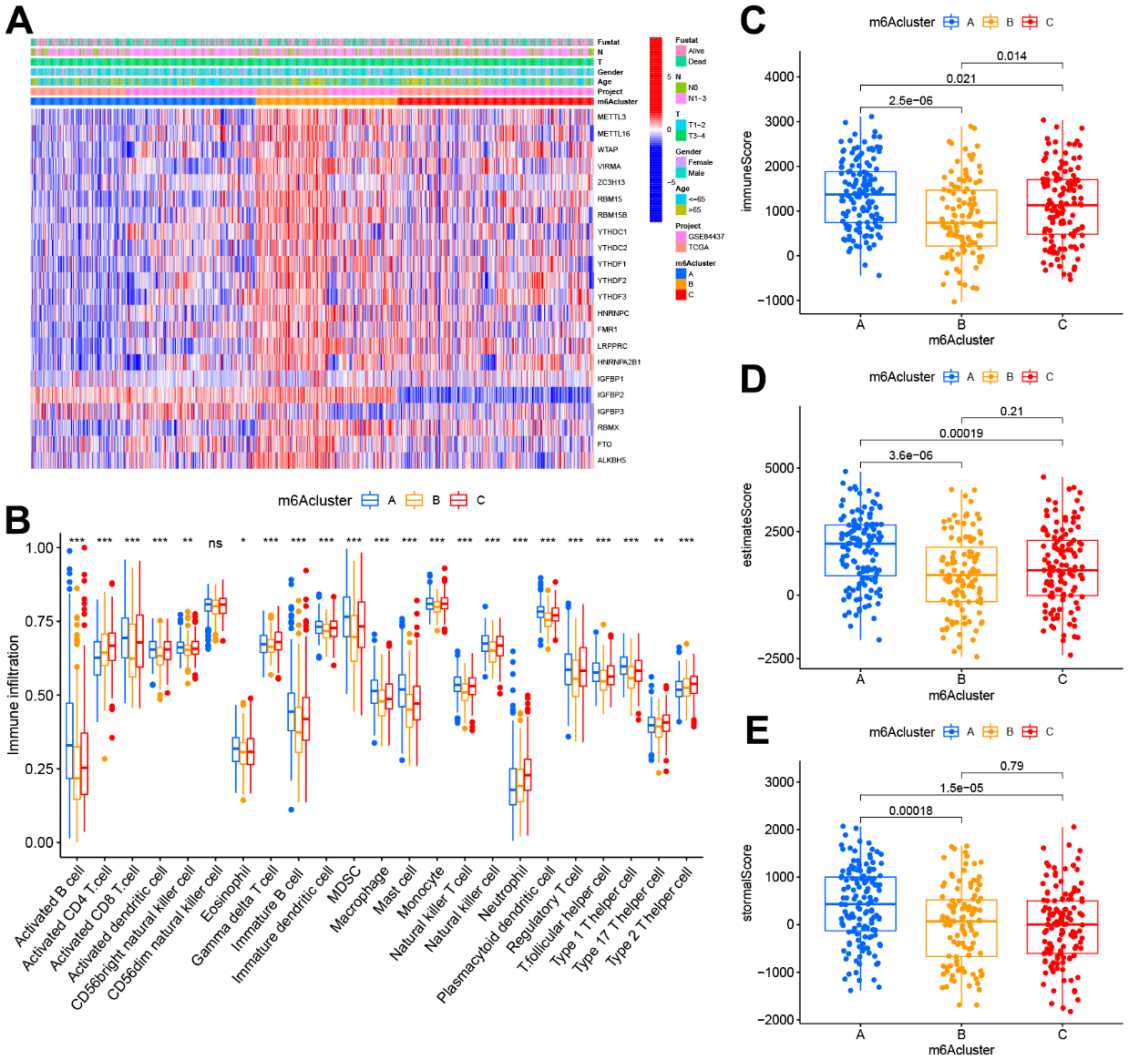
**TIME properties in the three m^6^A clusters.** (**A**) Thermogram depicting the consensus clustering outcome for the TCGA cohort, where age, gender, T&N stage, radiation, survival, and stage constituted the clinical traits. (**B**) CIBERSORT-based immunocyte infiltration in the three m^6^A clusters. *p < 0.05;**p < 0.01; ***p < 0.001. ns stood for no significance. (**C**–**E**) Assessment results of the (**C**) immune scores, (**D**) estimate scores, as well as (**E**) stromal scores across three m^6^A clusters.

We performed the heatmap and visualized the expression of 23 m^6^A regulators (Figure 4A) to investigate the correlation of these m^6^A clusters with clinical features. Most of the m^6^A regulators were up-expressed in cluster B, while IGFBP1 and IGFBP2 were down-regulated in cluster A. To our surprise, most people over 65 years significantly increased in cluster B. TIME and CIBERSORT package were applied to investigate the subsets of immune cells and feature the immune cell infiltration according to the expression file. Anti-tumor lymphocyte cells, like NK cells and activated CD8+ T cells, were primarily engaged in the m^6^A cluster A (Figure 4B). Cluster C was enriched with 2/17 T helper cells and gamma delta T cells. We demonstrated that m^6^A cluster A represented the highest immune scores, followed by m^6^A cluster C and m^6^A cluster B (Figure 4C). Accordingly, m^6^A cluster A displayed a higher tumor purity than m^6^A cluster C and m^6^A cluster B, suggesting that more stromal cells and immune cells surround tumors in m^6^A cluster C and B (Figure 4D). Previous studies had suggested a tumor stroma with multiple immune cells could retain these cells rather than the parenchyma of an immune-excluded phenotype.

### The m^6^A-related DEGs in STAD

**Figure 5:**
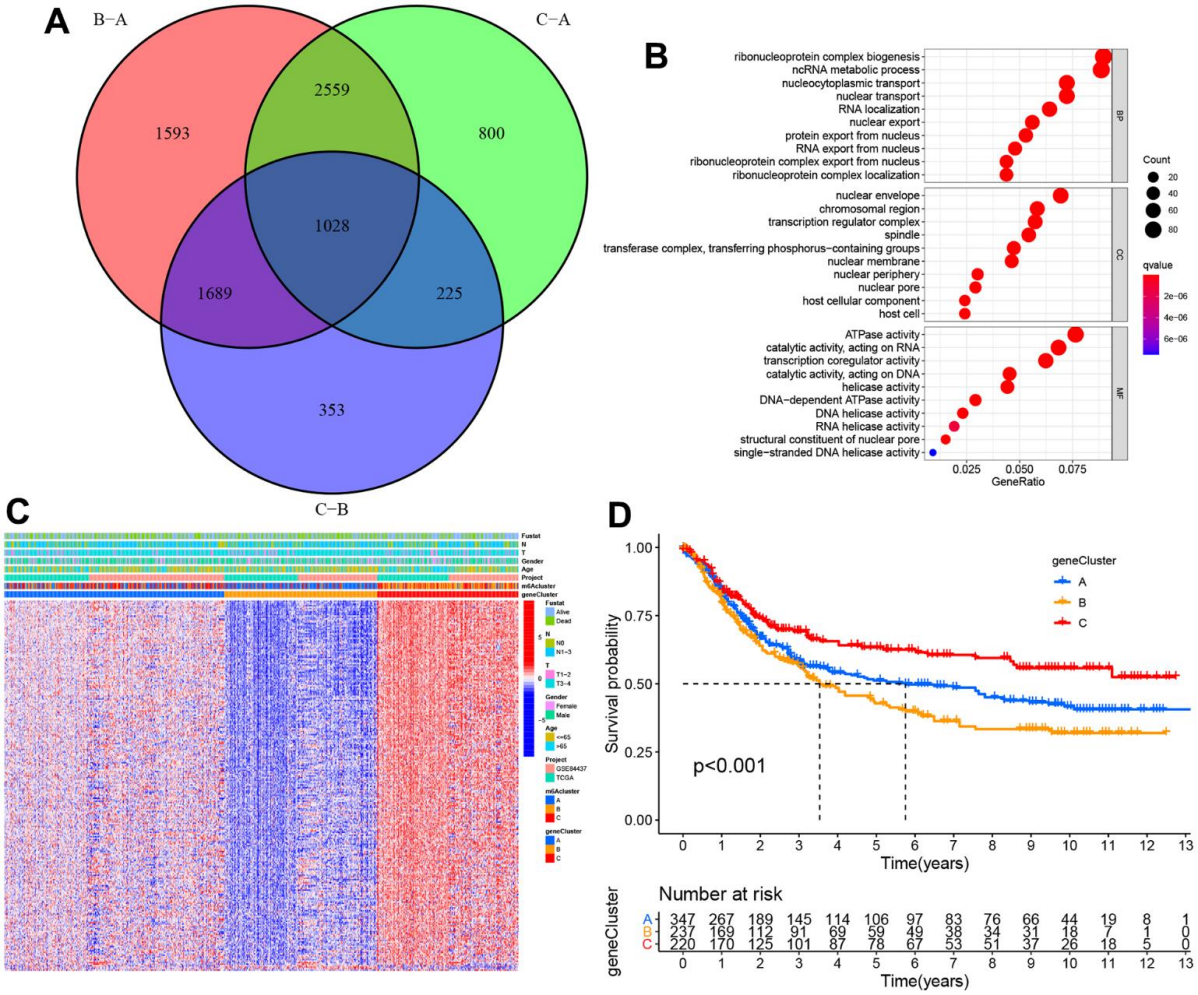
**The construction of m^6^A gene clusters and relevant functional annotations.** (**A**) Venn plot depicting 1,028 DEGs (differentially expressed genes) among three m^6^A clusters. (**B**) GO enrichment findings of 1,028 intersecting genes. (**C**) Intersect gene-based consensus clustering result of patients into three separate gene clusters. (**D**) Kaplan–Meier plots for the three m^6^A gene clusters (p < 0.001).

1018 DEGs were overlapped via the Venn diagram to identify the biological behaviors of these m^6^A clusters (e.g., expression perturbations and genetic alterations), and then, we examined transcriptional expression changes associated with three m^6^A clusters in STAD (Figure 5A). According to GO functional annotation, BPs associated with ribonucleoprotein complex biogenesis and RNA localization were significant bio-functions (Figure 5B). As per Figure 5C, three m^6^A gene clusters displayed distinct clinicopathological characteristics. Further, patients of late stages were mostly clustered into gene cluster C. Furthermore, all those three m^6^A gene clusters showed significantly different prognoses among the STAD samples based on survival analysis. Generally, samples of m^6^A cluster B had dismal prognostic outcomes, while those of m^6^A cluster C had superior prognostic outcomes (Figure 5D).

### Prognostic signature establishment and clinical feature exploration

According to those findings above, m^6^A regulators have essential effects on modulating TIME and prognostic outcome. However, the above analysis is based on the general population alone, whereas the heterogeneous and complicated m^6^A regulators are not explained separately. Based on those discovered m^6^A genes, this work established one scoring system referred to as m^6^A score to quantify scores of different individuals using those discovered m^6^A genes.

**Figure 6:**
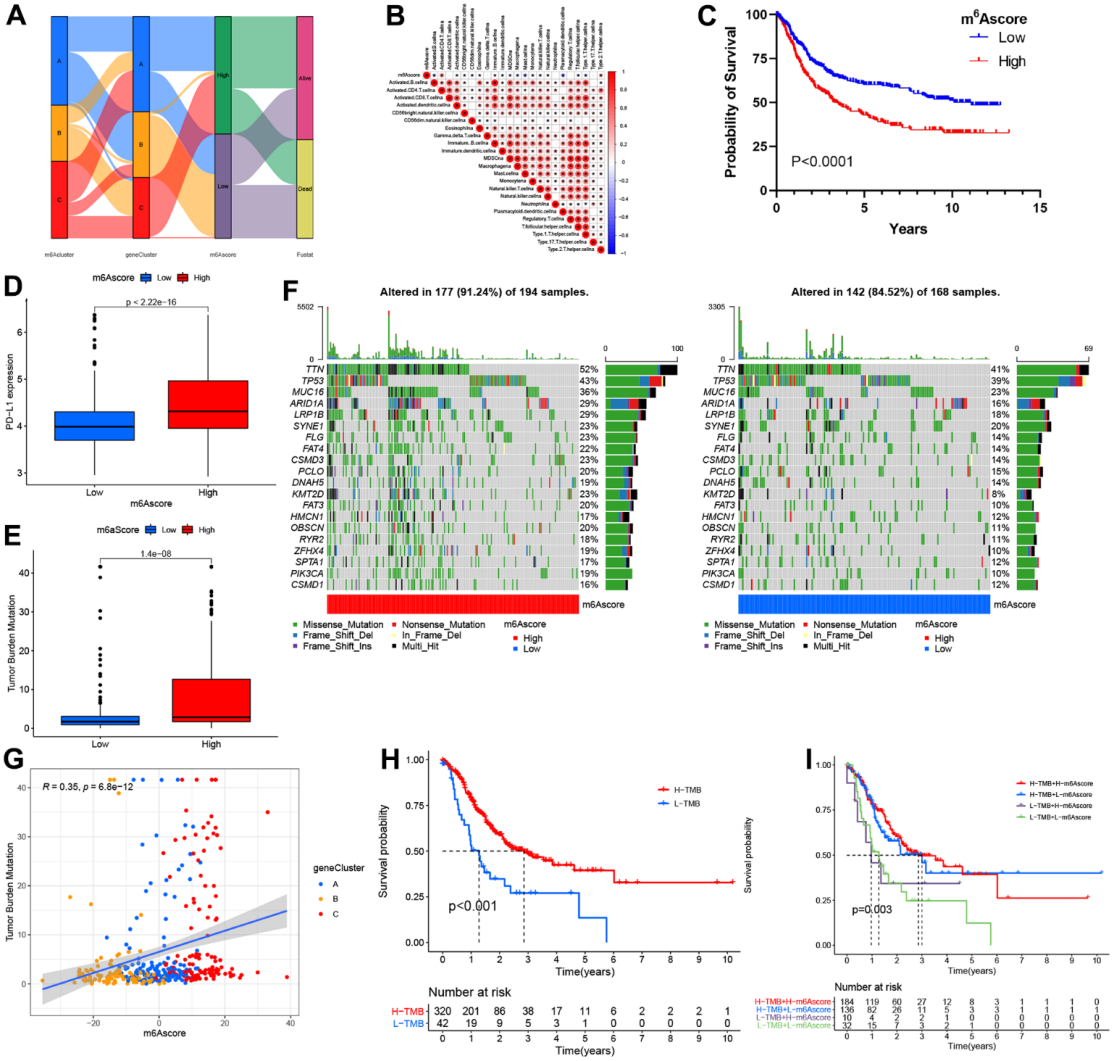
**The m^6^A score construction and relevant genetic trait assessment.** (**A**) Alluvial chart of m^6^A clusters regarding gene cluster and score of m^6^A, as well as patient survival. (**B**) Spearman correlations of m^6^A score with immunocytes. (**C**) Survival findings for the TCGA cohort patients marking high and low m^6^A scores. (**D**) PD-L1 level comparison between patients marking high and low m^6^A scores. (**E**) TMB (tumor mutation burden) distribution comparison between patients marking high and low m^6^A scores. (**F**) Mutational waterfall plot for the TCGA cohort patients marking high (left panel) and low (right panel) m^6^A scores. All patients were represented by individual columns. (**G**) Diagram illustrating significant positive association of m^6^A score with TMB (R = 0.35, p = 6.8–12). (**H**) Kaplan–Meier plots for patients exhibiting high (H) and low (L) TMBs. (P< 0.001). (**I**) Kaplan–Meier plots for the TCGA cohort patients stratified by both m^6^A score and TMB. H, high; L, Low; TMB, tumor mutation load (P = 0.003).

The alluvial diagram represents quantitative alterations in STAD samples (Figure 6A). These findings indicated that m^6^A clusters A/C showed increased m^6^A scores, while cluster B showed decreased m^6^A scores. In addition, we performed Spearman’s correlation analysis to illustrate m^6^A regulator patterns. Based on the heatmap, the m^6^A score showed immature B cell and activated CD4 T cell (Figure 6B). Next, this study evaluated the m^6^A score’s significance as a prognosis predictor for patients. We divided patients as low-or high m^6^A score subgroups as per the threshold. As expected, high m^6^A score patients were related to significantly poorer outcomes (Figure 6C). In this study, we also analyzed the relationship between m^6^A score and PD-L1 expression; as a result, high m^6^A score patients had increased PD-L1 expression compared to low m^6^A score patients (Figure 6D). Increasing evidence has illustrated that TMB is related to immunotherapeutic response. Thus, this work analyzed TMB distribution between high and low m^6^A score patients. According to our results, low m^6^A score patients were related to a decreased TMB frequency (Figure 6E). As shown in Figure 6G, the m^6^A score showed a marked positive correlation with TMB (R = 0.35, p = 6.8e-12). Further, we also examined somatic mutation gene distributions between both subgroups. According to Figure 6F, patients with high m^6^A scores had more somatic mutations than those with low scores.

Additionally, cases with increased TMB frequency had survival benefits (p<0.001; Figure 6H), whereas those with high m^6^A scores did not exhibit any difference in survival advantage between high and low TMB frequency (Figure 6I). Therefore, cases with an increased TMB frequency possibly gained more survival benefits from radiotherapy than those with a decreased frequency.

This work also examined the m^6^A score’s prognostic significance under diverse clinical factors. As a result, low m^6^A score cases were associated with superior prognosis to low m^6^A score patients from the T-stage subgroup (Supplementary Figure 5). Besides, low m^6^A score cases possibly gained more advantages from signatures than high m^6^A score cases.

### m^6^A score’s effect on estimating immunotherapeutic responses

**Figure 7:**
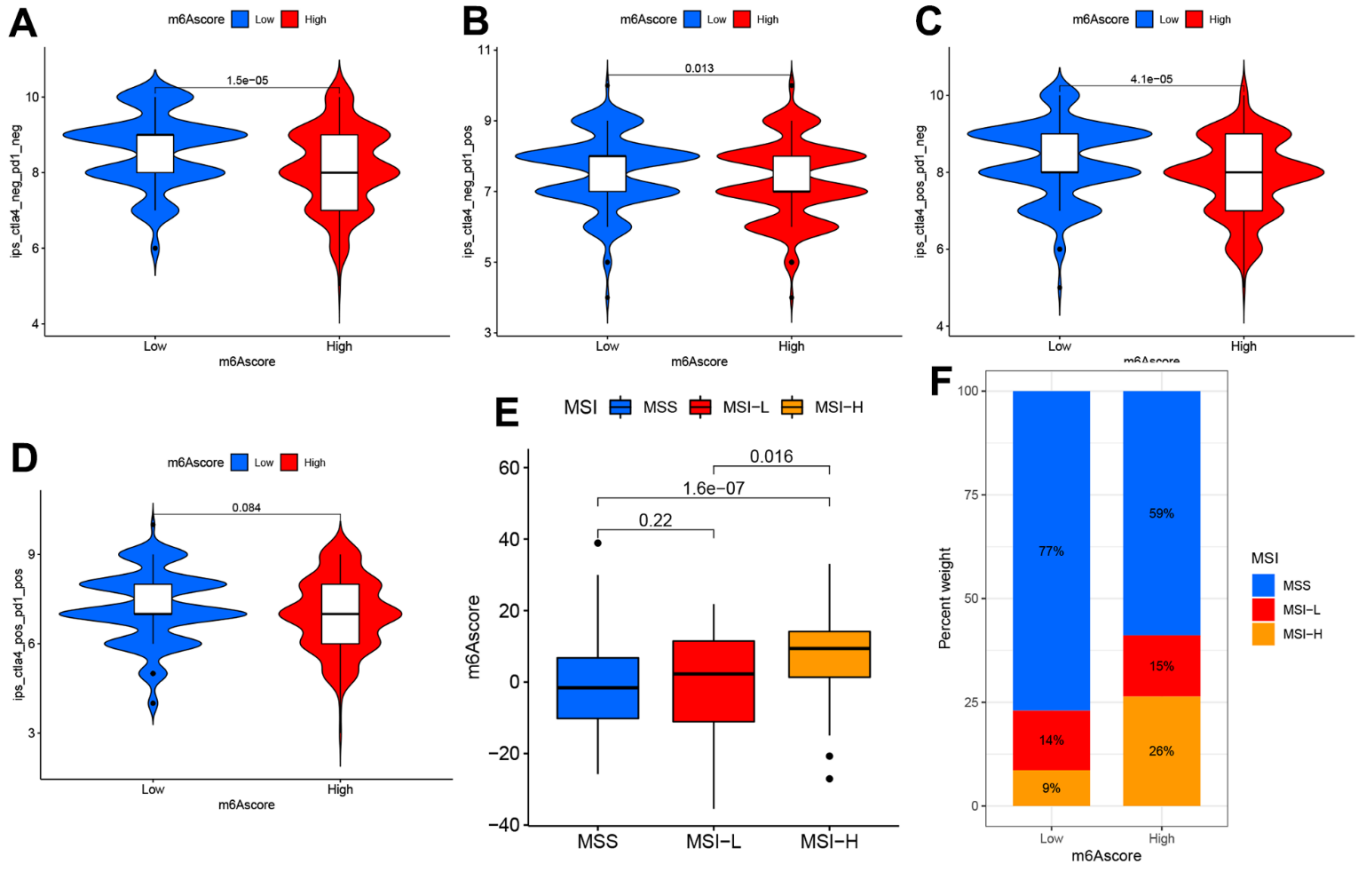
**The m^6^A score predicts immunotherapeutic benefits.** (**A**–**D**) TCIA database-based correlations of m^6^A score with IPS among the PAAD patients: (**A**) CTLA4(−) PD1(−) (**B**) CTLA4(−) PD1(+) (**C**) CTLA4(+) PD1(−) (**D**) CTLA4(+) PD1(+). (**E**) Distribution of m^6^A score in different MSI groups. (**F**) The ratio of MSI (microsatellite instability) in STAD samples with MSS and MSI-L and MSI-H.

This study examined differential expression of immune checkpoints (ICPs) to predict immunotherapeutic responses among STAD patients. IPS for the 140 TCIA- derived PAAD cases were identified as the favorable factor that predicted anti-cytotoxic T lymphocyte antigen-4 (CTLA-4) and anti-programmed cell death protein 1 (anti-PD-1) antibody responses, and it was significantly different between the two groups that they were both positive (Figure 7A–7D). Additionally, the other two immunotherapy cohorts, IMvigor210 cohort and GSE78220, were used to analyze the role of m^6^Acore in predicting anti-PD-L1 therapeutic response in patients. As for IMvigor210 and GSE78220 cohorts, patient survival did not consistently respond between low and high m^6^A score groups (Supplementary Figure 6). In conclusion, the results of this study suggest a close relationship between m^6^A regulators and TIME, which is responsible for immunotherapeutic responses.

### External validation of five key prognostic m^6^A RNA modulators

**Figure 8:**
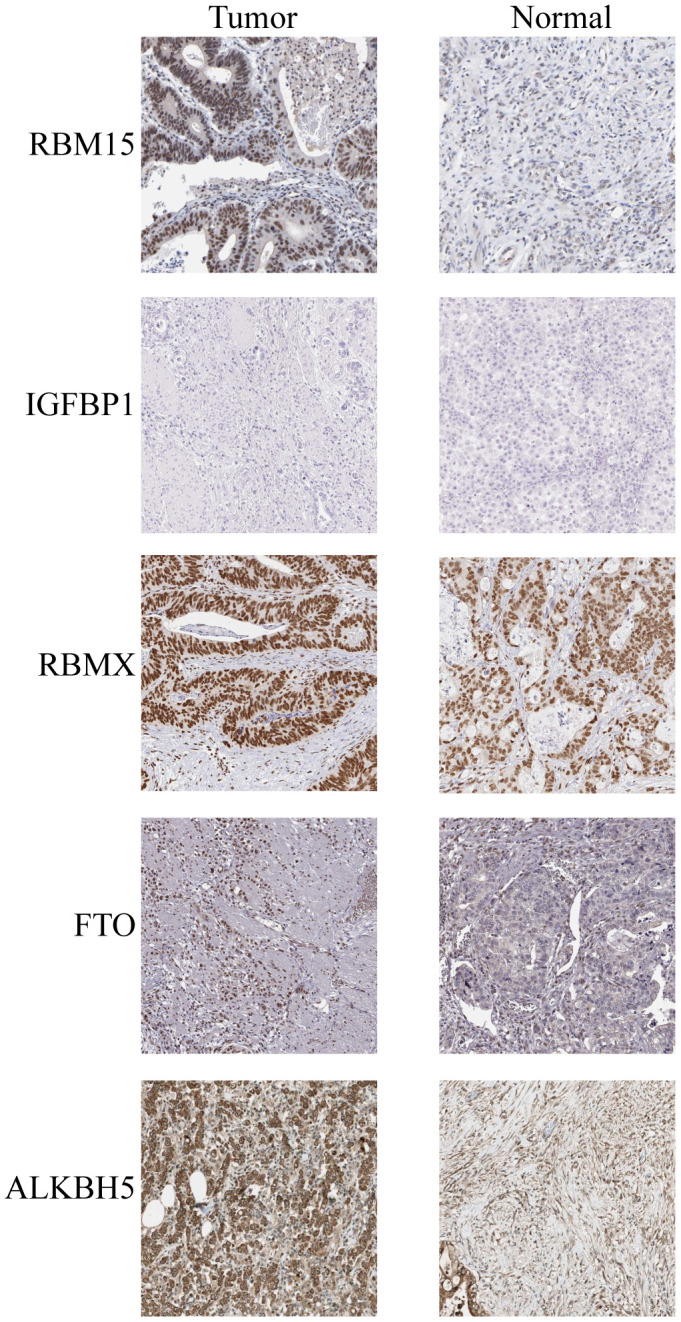
IHC analysis of five m^6^A modulators in stomach adenocarcinoma tissues and adjacent normal tissues.

IHC staining of five paired STAD and adjacent non-tumorous tissues was used to validate the protein expression of the five prognosis genes (Figure 8). The compared with adjacent non-tumorous tissues, we found a higher level of RBM15, IGFBP1, RBMX, FTO, and ALKBH5 in all five STAD tissues, RBM15, FTO and ALKBH5 were overexpressed in STAD tumor tissues, while the expression of IGFBP1, RBMX was not changed in STAD tumor tissues.

## DISCUSSION

There is growing evidence that m^6^A methylation, the most common post-transcriptional modification, regulates inflammation, immunity, and anti-tumor interactions with various m^6^A regulators. Additionally, as various articles illustrate only the impact of one or two regulators on TIME, it is required to comprehensively characterize several m^6^A regulator-mediated immune cells to understand how m^6^A methylation occurs within TIME. To date, the effect of m^6^A modulators on the TIME of STAD has not been fully understood. Determining the role of m^6^A modulators within TIME can elucidate the anticancer responses regulated by m^6^A methylation and contribute to developing effective chemotherapeutic and immunotherapeutic treatments.

This study constructed three immunophenotypes by adopting a total of 23 m^6^A regulators associated with STAD prognosis and distinct temporal characteristics. m^6^A cluster A had higher adaptive immune cell infiltration levels, indicating an immunoinflammatory phenotype. m^6^A cluster B was related to increased stromal cell and innate immunocyte infiltration degrees, corresponding to an immune-excluded phenotype. Similarly, m^6^A cluster C had the features of temporal suppression related to the immune-desert phenotype. This study indicates that immune-desert phenotypes lack primed and activated T cells, and this is related to immune evasion reported previously [20, 29, 30]. Moreover, m^6^A cluster A displayed a close association with a higher lymphocyte infiltration degree. Moreover, m^6^A cluster A displayed a close association with a higher lymphocyte infiltration degree, demonstrating that it might be used to predict immunotherapeutic response. Consequently, the m^6^A cluster A group possibly gained more survival benefits from ICB therapy. Overlapping DEGs identified from the three m^6^A phenotypes were closely related to immune pathways and RNA modification; consequently, such DEGs were the “real” m^6^A-related genes. Three transcriptome isoforms have also been discovered according to those identified m^6^A-related genes indicating their crucial role in shaping TIME. After that, this work constructed the m^6^A score scoring system for differentiating m^6^A modification-derived heterogeneity in individual cases to precisely guide the therapeutic strategies for STAD individuals. Our results showed that the immune-desert phenotype-featured m^6^A modification pattern showed increased m^6^A scores, whereas the immune-inflammatory phenotype-featured pattern had decreased m^6^A scores.

Furthermore, m^6^A scores might be adopted as prognostic markers, which were related to TMB and mutation-associated signatures. The m^6^A score served as the favorable prognostic biomarker for genomic aberrations based on the above findings.

A positive correlation was demonstrated between m^6^A scores and ICB therapeutic predictors, suggesting the potential influence of m^6^A methylation on the immunotherapy response among patients. Accuracy verification was conducted on the identified immunophenotype using the IMvigor210 cohort, which demonstrated that the m^6^A score in concert with multiple biomarkers (e.g., TMB, neoantigen load, the composition of TIME) was more effective in predicting the outcome of immunotherapy in patients [20]. We also utilized another two independent immunotherapy cohorts to perform prognostic power validation of m^6^A score in the immune response against PD-1/L1, and we further found that patients with high m^6^A anticancer drugs work better. Based on the above findings, the m^6^A score can be used to comprehensively identify immune-related phenotypes and guide clinical treatment decisions for immunotherapy and anticancer drugs.

In addition, the functionality of specific m^6^A regulators has been clarified for the TIME adjustment. In agreement with the latest research findings, m^6^A enhances mRNA stability and transports specific mRNAs into the cytoplasm mainly via the hnRNP A2/B1 binding protein in cells [31]. Additionally, hnRNP A2/B1 has been shown to play an oncogenic role in certain carcinomas, as evident from its facilitation of tumor development and migration [32–34]. Our analytical work noted up-regulation of hnRNP A2/B1 in tumors, exhibiting an association with survival reduction. Furthermore, high expression of hnRNP A2/B1 showed a lower tendency to infiltrate different types of DCs, s indicating that hnRNP A2/B1 might be involved in the DC initiation. Through analyzing the TCGA-STAD cohort, we also evaluated the mutant driver genes, key underpinnings of tumor diagnosis, and treatment options. Despite the addition of 23 m^6^A regulators in this mode, novel regulators still need to be picked to optimize the m^6^A score accuracy. Given the lack of an adequate immunotherapy cohort based on STAD, we expect various STAD regimens (e.g., anti-PD-1/L1 or anti-CTLA-4) will validate our conclusions. Besides, since the regulators and scores of m^6^A were identified by means of retrospective datasets, a prospective cohort of patients undergoing immunotherapy was required. Moreover, not all cohorts demonstrated that ICB therapy was conducive to the patients marking low m^6^A scores; thus, the model accuracy must be validated and modified based on a large sample of multi-center clinical populations in conjunction with more clinical traits.

In conclusion, we developed a comprehensive multi-cohort-based assessment of the m^6^A regulators’ TIME properties. Based on our comprehensive assessment, we conclude that m^6^A modification is key for tumor immunomodulation. The development of more efficacious and profound protocols of immunotherapy will be guided by a comprehensive evaluation of the m^6^A modifications in TIME.

## Supplementary Material

Supplementary Figures

Supplementary Table 1
