# Supplementary material for: Development a m6A regulators characterized by the immune cell infiltration in stomach adenocarcinoma for predicting the prognosis and immunotherapy response
Source: Aging (Albany NY). 2023 Mar 17;15(6):1944–63. doi: 10.18632/aging.204574 (PMC10085598; doi:10.18632/aging.204574)
Supplement: Supplementary Figures [file aging-15-204574-s001.pdf]

SUPPLEMENTARY FIGURES

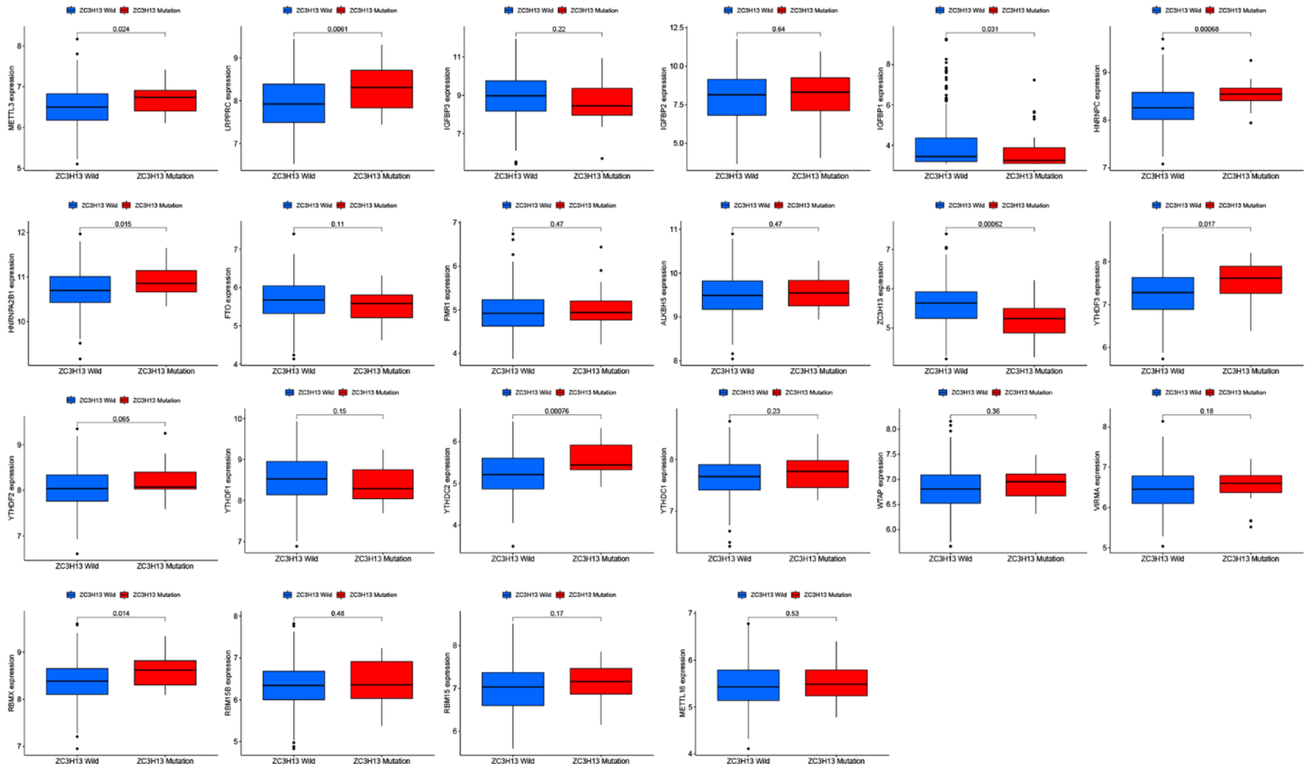

Supplementary Figure 1. Expression levels of 22 m^{6}a regulators between ZC3H13 mutant and wild-type in TCGA database.

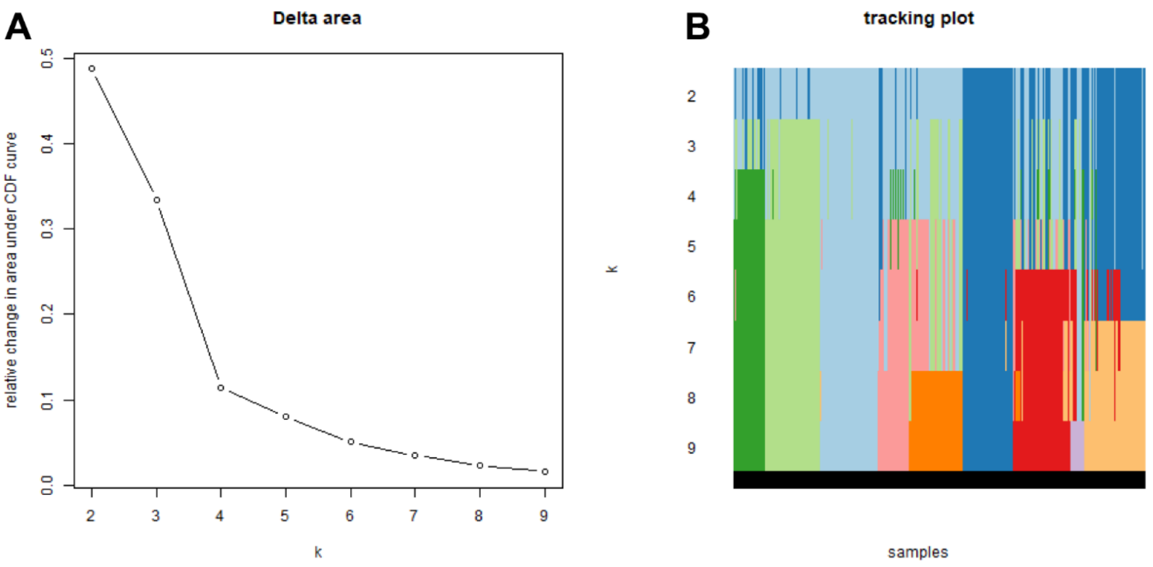

Supplementary Figure 2. Consensus clustering of 22 m^{6}A regulators in the TCGA cohort. (**A**) relative change in area under the CDF in left panel; (**B**) The cumulative distribution function (CDF) for k=2 to 9 in right panel; relative change in area under the CDF in left panel.

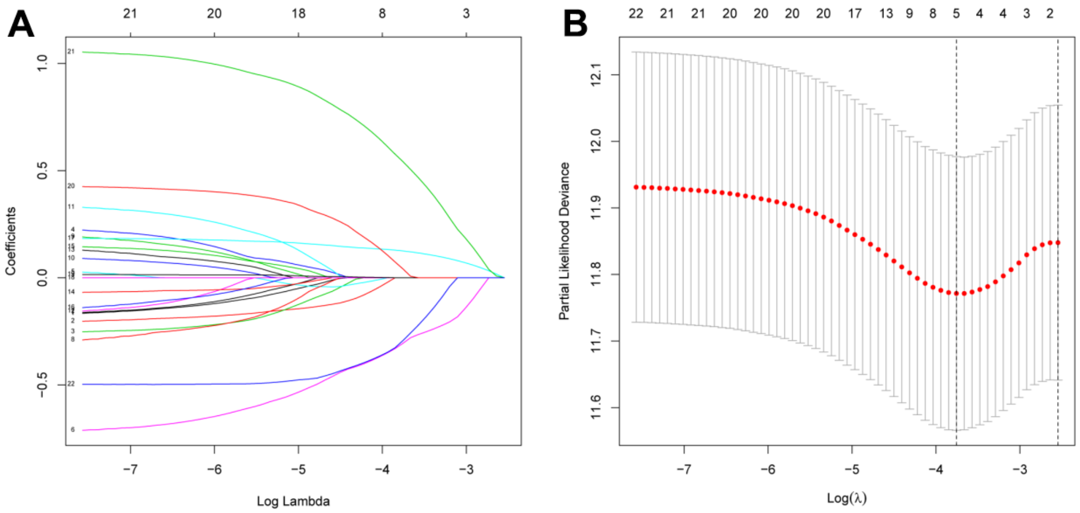

Supplementary Figure 3. Five selection of genes by LASSO Cox regression. (**A**) Selection of the optimal parameter (lambda) in the LASSO model for STAD. (**B**) LASSO coefficient profiles of the five genes in STAD. A coefficient profile plot was generated against the log (lambda) sequence.

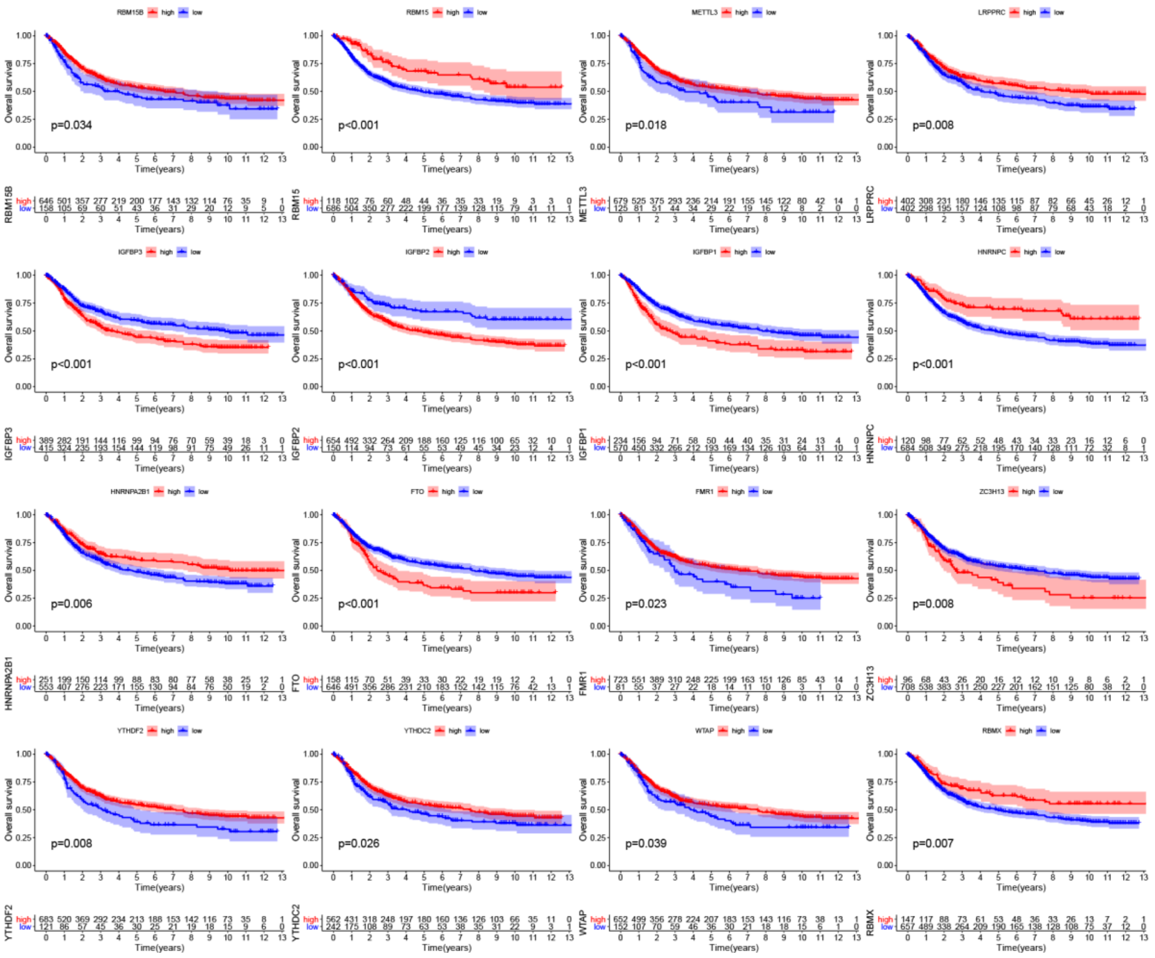

Supplementary Figure 4. The prognostic subgroup analysis of m^{6}A score based on the TCGA cohort.

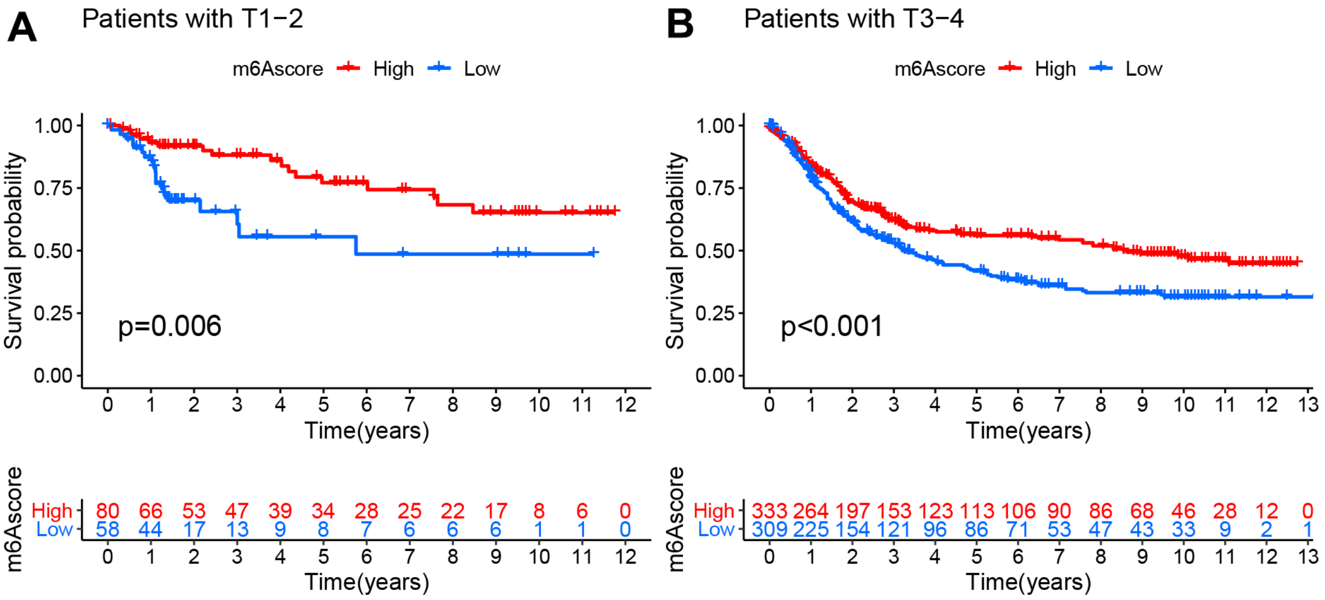

Supplementary Figure 5. The prognostic clinical status analysis of m^{6}A score based on the TCGA cohort. (**A**) K-M survival analysis in T1-2. (**B**) K-M survival analysis in T3-4.

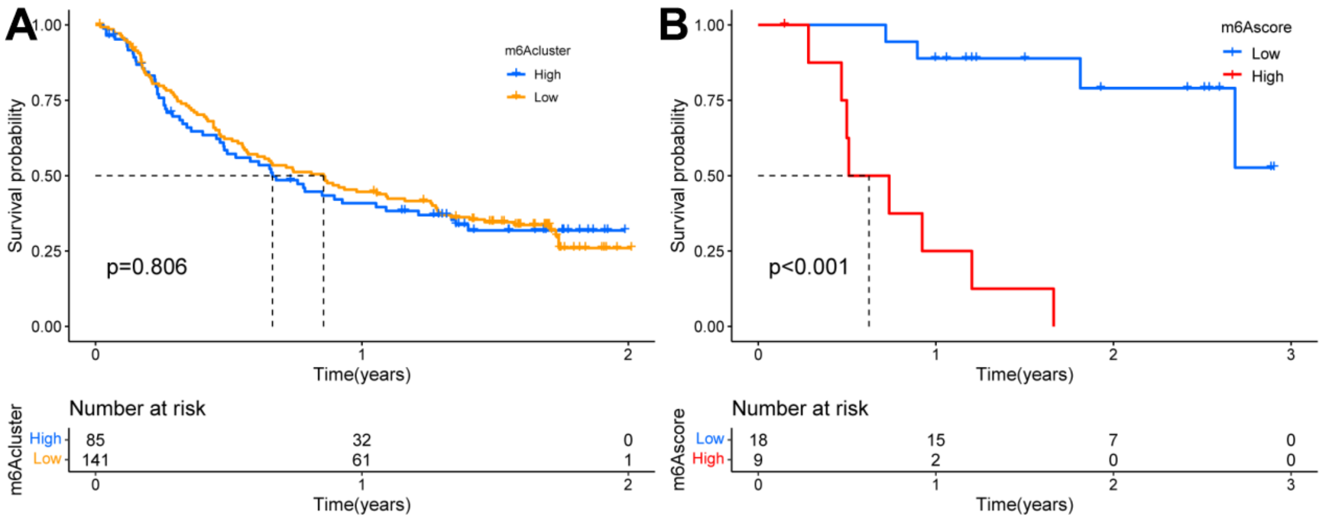

Supplementary Figure 6. The prognostic clinical status analysis of m^{6}A score between IMvigor210 and GSE78220 cohorts. (**A**) K-M survival analysis in high and low m6A cluster of IMvigor210. (**B**) K-M survival analysis in high and low m6A cluster of GSE78220.
