# Supplementary material for: Development a m6A regulators characterized by the immune cell infiltration in stomach adenocarcinoma for predicting the prognosis and immunotherapy response
Source: Aging (Albany NY). 2023 Mar 17;15(6):1944–63. doi: 10.18632/aging.204574 (PMC10085598; doi:10.18632/aging.204574)
Supplement: Supplementary Table 1 [file aging-15-204574-s002.pdf]

SUPPLEMENTARY TABLE

Supplementary Table 1. Methylated genotype.

Gene	Type
METTL3	writers
METTL14	writers
METTL16	writers
WTAP	writers
VIRMA	writers
ZC3H13	writers
RBM15	writers
RBM15B	writers
YTHDC1	readers
YTHDC2	readers
YTHDF1	readers
YTHDF2	readers
YTHDF3	readers
HNRNPC	readers
FMR1	readers
LRPPRC	readers
HNRNPA2B1	readers
IGFBP1	readers
IGFBP2	readers
IGFBP3	readers
RBMX	readers
FTO	erasers
ALKBH5	erasers
